# Exploring the impact of PEGylation on pharmacokinetics: a size-dependent effect of polyethylene glycol on prostate-specific membrane antigen inhibitors

**DOI:** 10.1186/s13550-024-01071-z

**Published:** 2024-02-07

**Authors:** Yang Liu, Li Xia, Haiyang Li, Ping Cai, Sufan Tang, Yue Feng, Guangfu Liu, Yue Chen, Nan Liu, Wei Zhang, Zhijun Zhou

**Affiliations:** 1 Department of Nuclear Medicine, The Affiliated Hospital, Southwest Medical University, Jiangyang District, Luzhou, Sichuan China; 2 Nuclear Medicine and Molecular Imaging Key Laboratory of Sichuan Province, Department of Nuclear Medicine, The Affiliated Hospital, Southwest Medical University, Jiangyang District, Luzhou, Sichuan China; 3 Institute of Nuclear Medicine, Southwest Medical University, Jiangyang District, Luzhou, Sichuan China; 4 Department of Pharmaceutics, School of Pharmacy, Southwest Medical University, Jiangyang District, Luzhou, Sichuan China; 5 Department of Nuclear Medicine, Sichuan Provincial People’s Hospital, University of Electronic Science and Technology of China, Sichuan, Chengdu China

**Keywords:** Prostate-specific membrane antigen (PSMA), Prostate cancer (PCa), Polyethylene glycol (PEG), LNCaP, Micro-PET/CT

## Abstract

**Background:**

Prostate cancer is the second most frequent cancer and the fifth leading cause of cancer-related deaths in men. Prostate-specific membrane antigen (PSMA) as a target has gained increasing attention. This research aims to investigate and understand how altering size of PEG impacts the in vitro and in vivo behavior and performance of PSMA inhibitors, with a specific focus on their pharmacokinetic characteristics and targeting properties.

**Results:**

Two ^68^Ga-labeled PSMA-targeted radiotracers were developed, namely [^68^Ga]Ga-PP4-WD and [^68^Ga]Ga-PP8-WD, with varying sizes of polyethylene glycol (PEG). [^68^Ga]Ga-PP4-WD and [^68^Ga]Ga-PP8-WD had excellent affinity for PSMA with IC50 being 8.06 ± 0.91, 6.13 ± 0.79 nM, respectively. Both tracers enabled clear visualization of LNCaP tumors in PET images with excellent tumor-to-background contrast. They also revealed highly efficient uptake and internalization into LNCaP cells, increasing over time. The biodistribution studies demonstrated that both radioligands exhibited significant and specific uptake into LNCaP tumors. Furthermore, they were rapidly cleared through the renal pathway, as evidenced by [^68^Ga]Ga-PP4-WD and [^68^Ga]Ga-PP8-WD showing a tenfold and a fivefold less in renal uptake, respectively, compared to [^68^Ga]Ga-Flu-1 in 30 min. Both in vitro and in vivo experiments demonstrated that PEG size significantly impacted tumor-targeting and pharmacokinetic properties.

**Conclusions:**

These radiotracers have demonstrated their effectiveness in significantly reducing kidney uptake while maintaining the absorbed dose in tumors. Both radiotracers exhibited strong binding and internalization characteristics in vitro, displayed high specificity and affinity for PSMA in vivo*.*

**Supplementary Information:**

The online version contains supplementary material available at 10.1186/s13550-024-01071-z.

## Introduction

Prostate cancer (PCa) is the second most frequent cancer and the fifth leading cause of cancer-related deaths in men [1]. The American Cancer Society estimated that there would be approximately 2.7 million new cases and 34,500 PCa-related deaths in 2022, and the numbers will grow to 2.9 million new PCa cases and 34,700 PCa-related deaths in 2023 [1, 2]. PCa mostly occurs at the primary site and has a good prognosis, but PCa is more likely to metastasize and recur, with a metastasis rate of 30–40% after treatment, eventually progress to castration-resistant prostate cancer (CRPC), which is the leading cause of death in PCa patients [3–5]. Despite significant efforts, currently available diagnostic and therapeutic strategies are often ineffective [6, 7]. Therefore, accurate diagnosis and grading of PCa are crucial for effective and successful patient treatment [8].

**Fig. 1:**
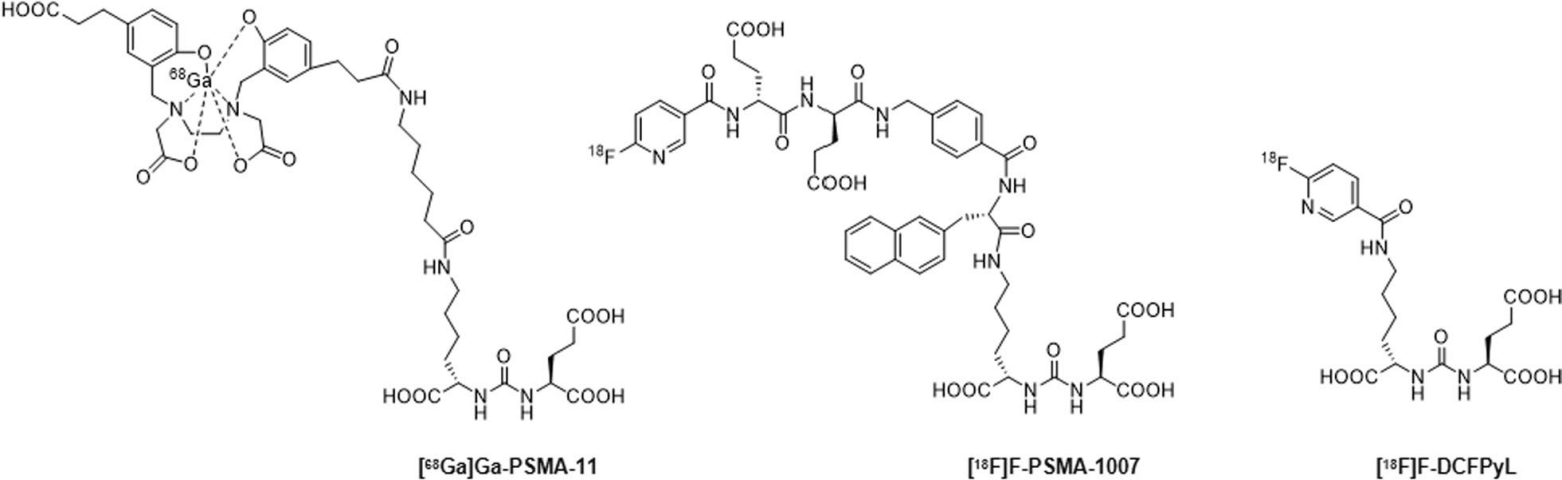
The most-often-used PET tracers for PCa detection are based on the Lys-urea-Glu scaffold

Recently, Positron emission tomography (PET) imaging with prostate-specific membrane antigen (PSMA) as a target has gained increasing attention. PSMA, also known as glutamate carboxypeptidase II (GCPII), is overexpressed in almost all types of human PCa as well as in neovascularization of various solid tumors and the expression level of PSMA increases with tumor grade and stage [9–12], but with significantly lower expression in healthy tissues [13, 14]. As a result, PSMA can serve as a target for PCa imaging and targeted therapy through binding of targeting molecules [15–20]. Despite the clinical success of certain radiotracers such as [^68^Ga]Ga-PSMA-11, [^18^F]F-PSMA-1007, and [^18^F]F-DCFPyL (Fig. 1), there is still a significant demand for molecules with exceptional specificity, affinity, and favorable pharmacokinetics, especially those possessing theranostic properties.

**Fig. 2:**
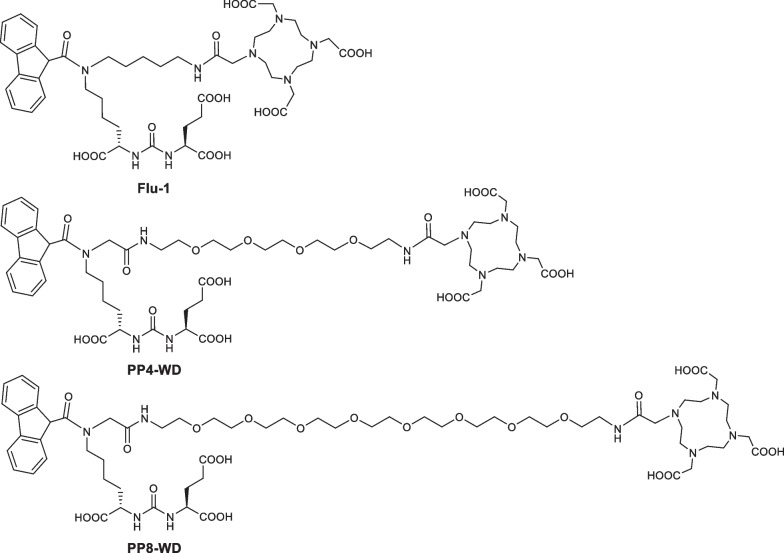
The chemical structures of Flu-1, PP4-WD, and PP8-WD

We previously reported a compound [^68^Ga]Ga-Flu-1, a lysine-ureido-glutamate-based PET tracer with DOTA as chelator (Fig. 2), bearing a lipophilic bulky group (9-carboxyfluorene) on the lysine residue. [^68^Ga]Ga-Flu-1 showed superior properties such as high tumor-to-background contrast, higher tumor uptake, and lower kidney uptake compared with [^68^Ga]Ga-PSMA-11. Despite reduced kidney uptake, this value still was 3 folds greater than that in the tumor at 2 h 21.High uptake by the kidneys might potentially lead to the failure to identify metastases in or near the kidneys [22]. In the past decades, researchers discovered that, attachment of PEG to peptides or proteins, so-called PEGylation, offers improved water solubility and stability as well as reduced clearance through the kidneys, leading to a longer circulation time [23–26]. The PEGylation strategy inspired us to synthesize novel variants of Flu-1 by incorporating different sizes of PEG onto Flu-1 structure, with an objective of examining the effects of varying PEG sizes on the in vitro and in vivo properties of Flu-1.

## Materials and methods

### Precursor synthesis

**Scheme 1:**
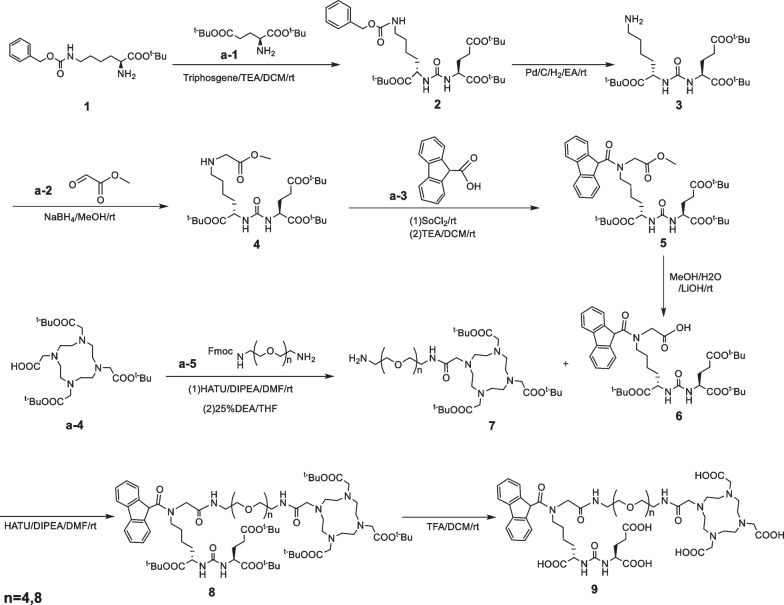
Synthesis of PP4-WD, PP8-WD. (PP4-WD: where n = 4, PP8-WD: where n = 8)

The synthetic routes and chemical structures of PP4-WD and PP8-WD were illustrated in Scheme 1. Both PP4-WD and PP8-WD were synthesized using multi-step reactions as reported by our group 27. The intermediate compound **3** has been reported somewhere else [21]. Subsequently, the precursors underwent purification through semi-preparative reversed-phase high-performance liquid chromatography (RP-HPLC). The comprehensive synthesis details are provided in the Supporting Information.

### ^***68***^***Ga radiolabeling***

^68^Ga as a positron-emitting isotope with a maximum energy of 1.9 MeV (88%), was obtained by eluting a ^68^Ge (*t*_1/2_ = 271 d)/^68^Ga (*t*_1/2_ = 68 min) generator (ITG, Germany) using a 0.05 M HCl solution. Radiolabeling of the compounds was performed by incubating 5–10 μg of the corresponding conjugate (1 mg/mL) with varying amounts of ^68^GaCl_3_ (18.5–40 MBq) in sodium acetate buffer (NaAc/HAc = 0.5 M/0.5 M) and heating the solution at 95 °C for 15 min. The reaction mixture was then diluted with 4 mL of saline and purified through a pre-activated Oasis HLB column, followed by washing with 5 mL of saline. The final product was eluted with 100 μL of 50% ethanol and diluted with 400 μL of physiological saline. The radiochemical purity was determined by RP-HPLC.

### ^***177***^***Lu-radiolabeling***

^177^Lu was provided by the Institute of Nuclear Physics and Chemistry at the China Academy of Engineering Physics (Mianyang, China). A quantity of ^177^LuCl_3_ (37–74 MBq) was transferred to a reaction vial containing 5–10 μg of the corresponding conjugate, along with 0.25 M sodium acetate buffer (NaAc/HAc = 0.5 M/0.5 M). The mixture was subjected to heat via vibration in a metal thermostatic bath at 95 °C for 15 min. Following this, the cooled reaction solution underwent filtration with sterile water using a pretreated Oasis HLB column. Radioactive purity was determined through RP-HPLC with 50% ethanol as the elution solvent, and the resulting solution was subsequently diluted with physiological saline.

### ^***nat***^***Ga-labeled standards***

To prepare ^nat^Ga-labeled standards, a solution of PP4-WD (0.59 mg, 0.5 μmol) or PP8-WD (0.68 mg, 0.50 μmol), was incubated with ultrapure ^nat^Ga(III)-chloride (Aladdin, China) (40 eq.) in 0.25 M sodium acetate buffer (NaAc/HAc = 0.5 M/0.5 M) (200 μL) and 0.05 M HCl (800 μL) at 95 °C for 15 min. The reaction mixture was then purified by RP-HPLC, and the RP-HPLC eluates containing the desired compound were collected, pooled, and lyophilized.

### Radiochemical stability

To investigate the stability of the ^68^Ga-labeled compounds, [^68^Ga]Ga-PP4-WD and [^68^Ga]Ga-PP8-WD were incubated in either phosphate-buffered saline (PBS) or human serum at 37 °C for 30, 60, and 120 min in a shaking incubator. The radiochemical purity of samples incubated in PBS at each time point was determined using RP-HPLC. For the samples in human serum, a pretreatment step was applied. Briefly, the human serum samples underwent precipitation with acetonitrile, and the radiochemical purity of each supernatant aliquot was determined using RP-HPLC after centrifugation for 5 min at 10,000 rpm. The experiments were performed in triplicate.

### Competitive cell binding assay

LNCaP prostate cancer cell line obtained from the American Type Culture Collection (ATCC, Manassas, VA) was used for cell affinity studies. The cells were grown in a meilunbio RPMI 1640 medium (ATCC modified) supplemented with 10% fetal bovine serum and 1% streptomycin/penicillin (Thermo Fisher Scientific, USA) at 37 °C in a humidified 5% CO_2_ atmosphere. Two days (48 ± 2 h) prior to in vitro experiments, the cells were harvested using trypsin-ethylenediaminetetraacetic acid (EDTA; 0.25% trypsin, 0.02% EDTA) in PBS and centrifuged. The supernatant was disposed, and the cell pellet was resuspended in a culture medium, and LNCaP cells (150,000 cells/well) were counted with a hemocytometer and seeded in poly-L-lysine-coated 24- well plates used in cell binding studies. The cells were then allowed to grow at 37 °C for 48 h. PC3-PIP cells provided by Professor Xiaoyuan Chen (Singapore) require additional Puromycin (2 µg/ml) in addition to the appealed culture conditions. Detailed information regarding uptake and internalization experiments can be found in the previous report [26].

In order to determine the binding affinity, a competitive cell binding assay was performed. LNCaP cells (100,000 cells/ well) seeded in 96-well plates were incubated with a 0.185 MBq/50μL solution of [^68^Ga]Ga-PSMA-11 in the presence of eight different concentrations of ^nat^Ga-PP4-WD or ^nat^Ga-PP8-WD (0 − 10,000 nM, 50 μL/well). After incubation for 1 h at 37 °C, the cells were washed with ice-cold PBS three times and lysed with 1 M NaOH. The total radioactivity in each well was measured with a gamma counter. The 50% inhibitory concentration (IC50) values were calculated by fitting the data using a nonlinear regression algorithm (GraphPad Prism Software). Experiments were performed at least three times including quadruplicate sample measurements.

### ***Log D***_***7.4***_

10 µL of each ^68^Ga-radiolabeled compound (~ 0.037 MBq) were added to a vial containing 500 µL of octanol and 490 µL of 0.01 M PBS (pH = 7.4). After vortexed for 5 min and centrifuging for 10 min (5000 rpm), the radioactive count of the octanol and PBS phases were determined with a γ-counter (CAPRAC-t, Edmonton, Canada). Log D_7.4_ was then determined using the following equation: Log D_7.4_ = Log [(γ counts in the octanol phase − *γ* counts in background)/(*γ* counts in PBS − γ counts in background)]. Each group was repeated 3 times, and the average value was expressed as log D_7.4_ ± standard deviation (SD).

### Biodistribution and imaging studies

All animal experiments were performed with the approval of the institutional animal ethics committee. Male NOD/SCID mice (5 − 6 weeks old) implanted with LNCaP cells were used for imaging and biodistribution experiments as previously described [27]. The mice were imaged or used in biodistribution studies once the tumor grew to 8 − 10 mm in diameter over a period of 4 − 5 weeks. At the same time, male balb/c-nu mice (5 − 6 weeks old) implanted with PC3-PIP cells were used as an alternative tumor model for imaging and biodistribution experiments.

To perform imaging studies, the male mice bearing LNCaP tumors were injected with the corresponding radioligand (~ 2.5 MBq; 100 μL) via their tail veins. The micro-PET/CT scans (Inveon PET, Siemens) were conducted at 10, 30, 60, and 120 min after injection. The mice were anesthetized and maintained under 2% isoflurane in oxygen at a flow rate of 2 L/min during the 2-h imaging study. First, a 10 min static PET imaging acquisition was carried out, followed by a 10 min CT scan for localization and attenuation correction. Data analysis was performed using Inveon Research Workplace software. For PC3-PIP tumor model, the imaging studies were performed with micro-PET/SPECT/CT (Inliview-3000B, Novel Medical). Data analysis was performed using Nmsoft-Al ws v1.7–1 software.

To conduct biodistribution studies, male mice bearing LNCaP or PC3-PIP tumors with an average body weight of approximately 20 ± 5 g and a tumor diameter of 8 − 10 mm were administered a bolus injection of 2.5 MBq of the corresponding radioligand via the tail vein. After 30, 60, and 120 min, the mice were anesthetized with isoflurane and subsequently euthanized by CO_2_ asphyxiation. Blood was drawn, and the organs of interest were promptly harvested, blotted dry, and weighed. The radioactivity of the collected mouse organs was measured and expressed as the percentage of the injected dose per gram of tissue (%ID/g). Each group consisted of at least five mice.

## Results

### Chemical and radiochemical synthesis and characterization

**Fig. 3:**
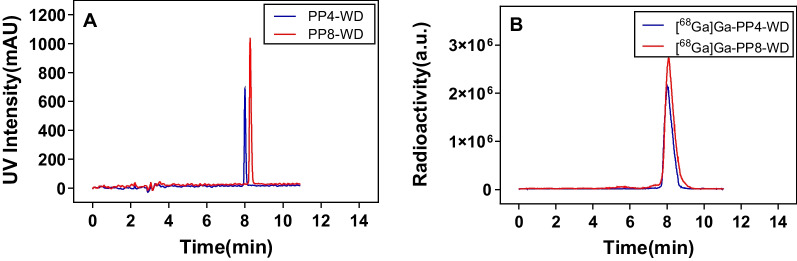
HPLC chromatogram of PP4-WD, PP8-WD (**A**) and Radio-HPLC chromatogram of [^68^Ga]Ga-PP4-WD, [^68^Ga]Ga-PP8-WD (**B**)

As shown in Scheme 1, the synthesis of these precursors through multiple step reactions is quite straightforward. We first constructed urea-based compound **2** bearing protected glutamate and lysine residues, followed by hydrogenation of compound **2** to yield compound **3**. Next, compound **3** underwent nucleophilic addition reaction with methyl glyoxylate, forming an imine, which was then reduced by NaBH_4_ to provide compound **4**. Compound **5** was obtained by reacting **4** with 9-carboxyfluorene, then the methyl group was removed to yield compound **6**. The conjugation of compound **6** with the DOTA chelator was achieved through an amidation reaction, followed by the removal of the Fmoc-protective group under alkaline conditions to obtain compound **7**. Subsequently, compounds **6** and **7** were subjected to an amidation reaction followed by deprotection in trifluoroacetic acid. Finally, the target molecule was purified using RP-HPLC, resulting in a purity of over 95% for both precursors. PP4-WD and PP8-WD were characterized by ESI + Mass and had retention times at 8.0 min and 8.3 min on RP-HPLC, respectively (Additional file 1: Fig. S1–S2, Fig. 3A).

### ^***68***^***Ga labeling***

The synthesis of ^68^Ga-labeled PSMA inhibitors was achieved by reacting PP4-WD or PP8-WD with ^68^GaCl_3_ in NaAc/HAc (v/v = 1/1 with pH = 4.3) buffer solution within 15 min at 95 °C. ^68^Ga labeling efficiency of both precursors analyzed with RP-HPLC for both ^68^Ga-labeled PSMA inhibitors are > 95%. After purification with Oasis HLB 1 cc (10 mg) extraction cartridges (Waters, USA), the radiochemical purity (RCP) for both radioligands then exceeded 98%. The retention times for [^68^Ga]Ga-PP4-WD and [^68^Ga]Ga-PP8-WD were 8.0 min and 8.1 min, respectively (Fig. 3B).

### Lipophilicity

Hydrophilicity of these radioligands were investigated by measuring the partition coefficient (Log D_7.4_) between octane and PBS. The Log D_7.4_ values of [^68^Ga]Ga-PP4-WD and [^68^Ga]Ga-PP8-WD were − 3.06 ± 0.15 and − 4.27 ± 0.26, respectively (Table 1). These results indicate that [^68^Ga]Ga-PP8-WD is more hydrophilic than [^68^Ga]Ga-PP4-WD.

**Table 1:** Analytical data of PP4-WD, PP8-WD, and Flu-1

Compound	Chemical formula	Calculated mass	m/z^1^	tr (min)^2^	Radiochemical purity (%)^3^	Log D_7.4_
PP4-WD	C_54_H_79_N_9_O_20_	1174.27	1174.55	8.01	97.98 ± 0.56	− 3.06 ± 0.15
PP8-WD	C_62_H_95_N_9_O_24_	1350.48	1350.66	8.09	96.56 ± 0.32	− 4.23 ± 0.26
Flu-1^4^	C_47_H_66_N_8_O_15_	983.43	983.47	8.13	88.53 ± 1.21	− 2.64 ± 0.25

^1^Mass spectrometry data detected as [M + H]^+^. ^2^Retention times of the Ga-labeled compounds.^3^Values of radiochemical purity were measured by RP-HPLC. An Agilent analytical column (250 × 4.6 mm) was utilized with mobile phases consisting of 0.1% TFA in water (A) and ACN (B). A linear gradient of solvent A (90–10% in 15 min) in solvent B (10–90% in 15 min) at a flow rate of 1.0 mL/min. ^4^Data for [^68^Ga]Ga-Flu-1 was obtained from a previously published report [21]

### Stability

**Fig. 4:**
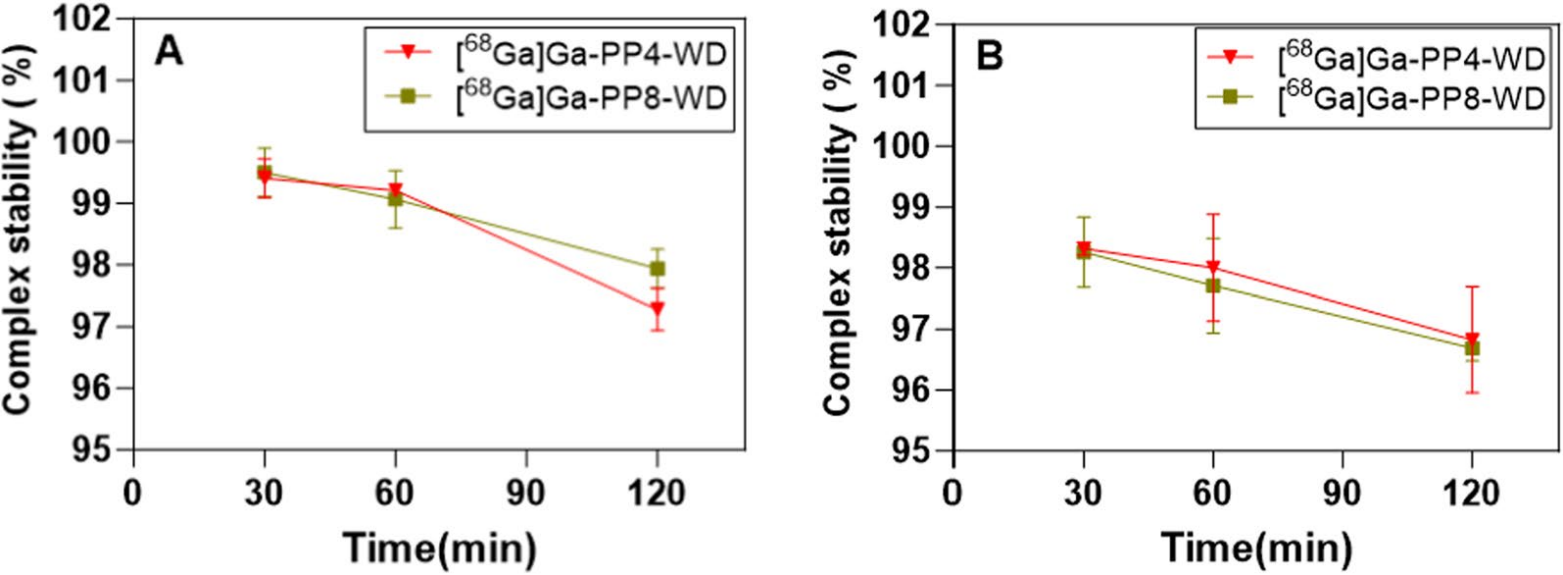
Stability of [^68^Ga]Ga-PP4-WD and [^68^Ga]Ga-PP8-WD. Radiochemical purity was recorded in PBS (**A**) and human serum (**B**) at 30, 60, and 120 min

The stability of both [^68^Ga]Ga-PP4-WD and [^68^Ga]Ga-PP8-WD was investigated by incubating each radioligand in either PBS or human serum at 37 °C (Fig. 4). After 2 h of incubation, the radiochemical purity of two radiotracers was slightly reduced in the PBS medium but still remained as high as 97%. Both radiotracers demonstrated remarkable stability in human serum, as indicated by the radiochemical purity of [^68^Ga]Ga-PP4-WD and [^68^Ga]Ga-PP8-WD remaining at 96.83 ± 0.87% and 96.69 ± 0.21% at 2 h, respectively.

### Cell affinity studies

**Fig. 5:**
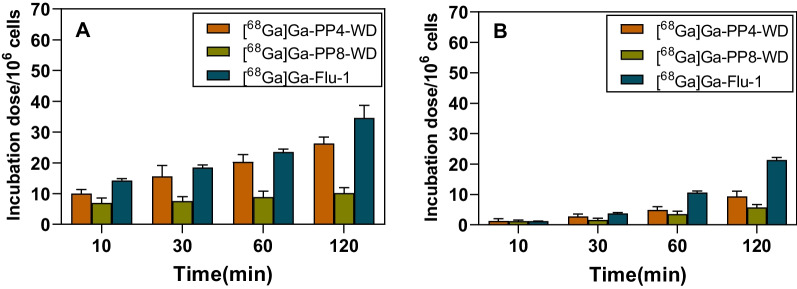
The uptake (**A**) and internalization (**B**) of [^68^Ga]Ga-PP4-WD, [^68^Ga]Ga-PP8-WD, and [^68^Ga]Ga-Flu-1 in LNCaP cells (~ 240,000 cells/well, normalized to 10^6^ cells) at 10, 30, 60, and 120 min

The specific cell surface binding and internalization into LNCaP cells were determined for [^68^Ga]Ga-PP4-WD and [^68^Ga]Ga-PP8-WD with [^68^Ga]Ga-Flu-1 as a reference. As shown in Fig. 5, both uptake and internalization of three radioligands displayed a time-dependent pattern and rose over 120 min duration. Specifically, the uptake and internalization of [^68^Ga]Ga-PP4-WD reached 26.30 ± 2.06% and 9.36 ± 1.70% after 120 min of incubation, respectively. Under the same condition, [^68^Ga]Ga-PP8-WD exhibited only moderate uptake and internalization rates, measuring at 10.16 ± 1.87% and 5.72 ± 0.95%, respectively, with a gradual increase observed over the same period. In contrast, [^68^Ga]Ga-Flu-1 demonstrated rapid enhancement in both uptake and internalization levels throughout the course of the experiments and eventually reached 34.57 ± 4.14% and 21.3 ± 0.13%, respectively, at 120 min. Overall, all three radioligands displayed increasing uptake and internalization levels over the course of experiments. Compared to the other two radioligands under the same conditions, [^68^Ga]Ga-Flu-1 revealed higher uptake and internalization levels.

The binding affinity of [^68^Ga]Ga-PP4-WD and [^68^Ga]Ga-PP8-WD was measured by in vitro competition binding assays using PSMA-expressing LNCaP cells and [^68^Ga]Ga-PSMA-11 as the reference compound. As shown in Additional file 1: Figure S3, both compounds competitively inhibited binding with [^68^Ga]Ga-PSMA-11 to LNCaP cells in a dose-dependent manner. The calculated IC_50_ values for [^68^Ga]Ga-PP4-WD, [^68^Ga]Ga-PP8-WD, and [^68^Ga]Ga-Flu-1 were 8.06 ± 0.91, 6.13 ± 0.79, and 9.62 ± 1.70 nM [20], respectively.

### Biodistribution

**Fig. 6:**
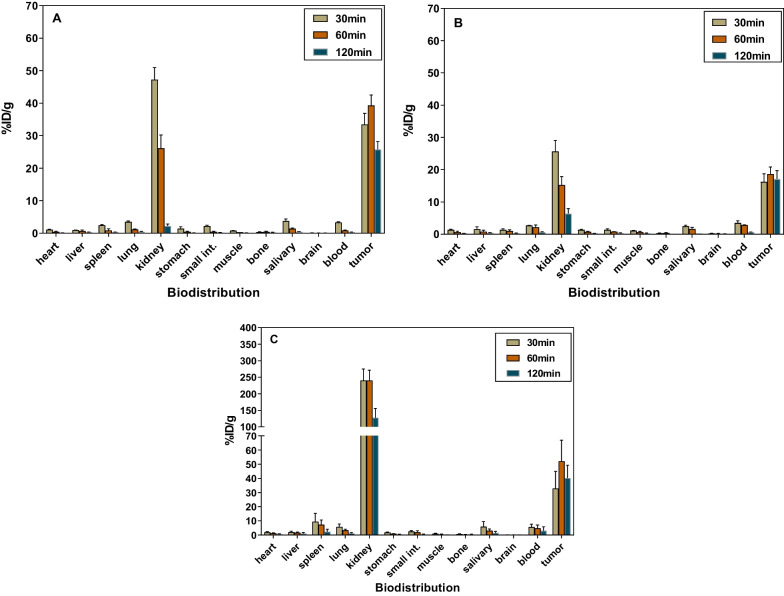
Organ biodistribution of [^68^Ga]Ga-PP4-WD (**A**), [^68^Ga]Ga-PP8-WD (**B**), and [^68^Ga]Ga-Flu-1 (**C**) in LNCaP tumor model expressed as %ID/g tissue at 30, 60, and 120 min post-injection (p.i.) Data are expressed as the mean ± SD (n = 5). small int. = small intestine

Biodistribution was conducted to evaluate the major organ distribution profile of radiotracers in LNCaP tumor-bearing NOD/SCID mice. [^68^Ga]Ga-Flu-1 was examined as the positive control, which was reported by our group previously [21]. The results were decay-corrected, listed as a percentage of the injected activity per gram of tissue mass (%ID/g), and presented as the average ± standard deviation (SD) (Fig. 6, Additional file 1: Tables S1–S3).

The results indicated that all radioligands exhibited high specific uptake and rapid accumulation in LNCaP tumors. After 30 min, the radioactivity accumulation of the three radioligands, namely [^68^Ga]Ga-PP4-WD, [^68^Ga]Ga-PP8-WD, and [^68^Ga]Ga-Flu-1, was found to be 33.45 ± 3.40%ID/g, 16.18 ± 2.53%ID/g, and 32.86 ± 12.02%ID/g, respectively. Furthermore, tumor uptake continued to increase over time, as demonstrated by the values of 39.28 ± 3.25%ID/g, 18.64 ± 2.20%ID/g, and 52.07 ± 14.83%ID/g at 60 min. However, these values decreased to 25.75 ± 2.43%ID/g, 17.12 ± 2.57%ID/g, and 40.11 ± 9.24% ID/g at 120 min.

The results showed that renal pathway is the primary route of excretion for all three radioligands. Specifically, the renal uptake of [^68^Ga]Ga-PP4-WD and [^68^Ga]Ga-PP8-WD was significantly reduced compared to [^68^Ga]Ga-Flu-1. The uptake values at 30 min were 47.24 ± 3.68%ID/g for [^68^Ga]Ga-PP8-WD and 25.63 ± 3.46%ID/g for [^68^Ga]Ga-PP4-WD, and 240.00 ± 34.68%ID/g for [^68^Ga]Ga-Flu-1. While the accumulated activity in kidneys decreased over time for all three radioligands, it remained relatively high for [^68^Ga]Ga-Flu-1 at 127.83 ± 27.94%ID/g, in contrast, there was a substantial reduction in accumulated activity for both [^68^Ga]Ga-PP4-WD and [^68^Ga]Ga-PP8-WD, with values of 2.23 ± 0.58%ID/g and 6.39 ± 1.56%ID/g, respectively. For other normal organ/tissues, the radioactivity accumulated was rapidly eliminated.

In contrast to the biodistribution results of the LNCaP model, [^68^Ga]Ga-PP4-WD and [^68^Ga]Ga-PP8-WD tumors were slightly decreased in the PC3-PIP tumor model. The uptake of [^68^Ga]Ga-PP4-WD and [^68^Ga]Ga-PP8-WD in tumors was 27.43 ± 1.81%ID/g and 15.21 ± 3.33%ID/g, respectively, compared to the uptake values of 39.28 ± 3.25%ID/g and 18.64 ± 2.20%ID/g in LNCaP tumor mice model, respectively. However, the trend of tumor uptake at each time point was the same as in the LNCaP model, such that although renal uptake of [^68^Ga]Ga-PP4-WD was higher than [^68^Ga]Ga-PP8-WD at 30 min, renal uptake of [^68^Ga]Ga-PP4-WD was lower than [^68^Ga]Ga-PP8-PD WD after 60 min and 120 min. In the PC3-PIP model, the peak uptake was still around 60 min, while the tumor uptake of [^68^Ga]Ga-PP4-WD was also higher than the [^68^Ga] Ga-PP8-WD at the corresponding time points, which is consistent with the characteristics in the LNCaP model. In addition, according to the biodistribution results of the PC3-PIP model, the overall uptake of [^68^Ga]Ga-PP8-WD was increased slightly in non-target organs, but these increases were all small or even negligible (Additional file 1: Figure S4, Tables S7–S8).

### Tumor-to-normal tissue (T/N)

**Fig. 7:**
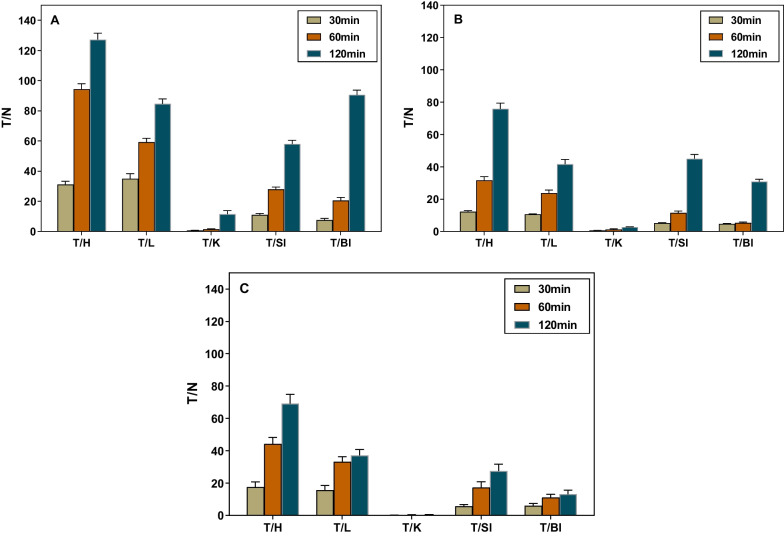
The tumor-to-heart (T/H), tumor-to-liver (T/L), tumor-to-kidney (T/K), tumor-to-salivary (T/Sl) and tumor-to-blood (T/Bl) values at 30, 60, and 120 min were obtained from the biodistribution data of [^68^Ga]Ga-PP4-WD (**A**), [^68^Ga]Ga-PP8-WD (**B**), and [^68^Ga]Ga-Flu-1 (**C**) in LNCaP tumor model

The biodistribution data in LNCaP tumor model at 30, 60, and 120 min were used to calculate the ratios of tumors to key normal organs (Fig. 7, Additional file 1: Tables S4–S6). As illustrated in Fig. 7, within a two-hour time course, the ratios for target organs exhibited a consistent upward trend for all three radioligands. Interestingly, the data indicated that while the tumor uptake of [^68^Ga]Ga-PP4-WD is lower than that of [^68^Ga]Ga-Flu-1, the T/N ratios for [^68^Ga]Ga-PP4-WD in all selected organs are significantly higher than that of both [^68^Ga]Ga-PP8-WD and [^68^Ga]Ga-Flu-1.

### Micro-PET/CT imaging

NOD/SCID mice bearing LNCaP tumors were selected for the whole-body micro-PET/CT imaging study of [^68^Ga]Ga-PP4-WD, [^68^Ga]Ga-PP8-WD, and the reference radiotracer [^68^Ga]Ga-Flu-1.

To evaluate the specificity of radioligands, blocking experiments were performed. In brief, 40 nmol of the PSMA inhibitor 2-PMPA was administered, followed by the injection of approximately 2.6 MBq of radioligands after 30 min. Then a static scan of micro-PET/CT was performed 60 min later. Upon blocking, it was observed that there was substantially reduced radioactivity detected for both [^68^Ga]Ga-PP4-WD and [^68^Ga]Ga-PP8-WD (Additional file 1: Figure S5A, S5B). Meanwhile, no significant reduction in uptake within normal organs, indicating the exceptional specificity of [^68^Ga]Ga-PP4-WD and [^68^Ga]Ga-PP8-WD for LNCaP tumors. In parallel, we conducted blocking imaging of PC3-PIP using the identical methodology as previously described, and the outcomes were in concordance with those observed in the LNCaP tumor model (Additional file 1: Figure S5C, S5D). This consistency underscores the remarkable specificity of [^68^Ga]Ga-PP4-WD and [^68^Ga]Ga-PP8-WD for PSMA-positive tumors.

**Fig. 8:**
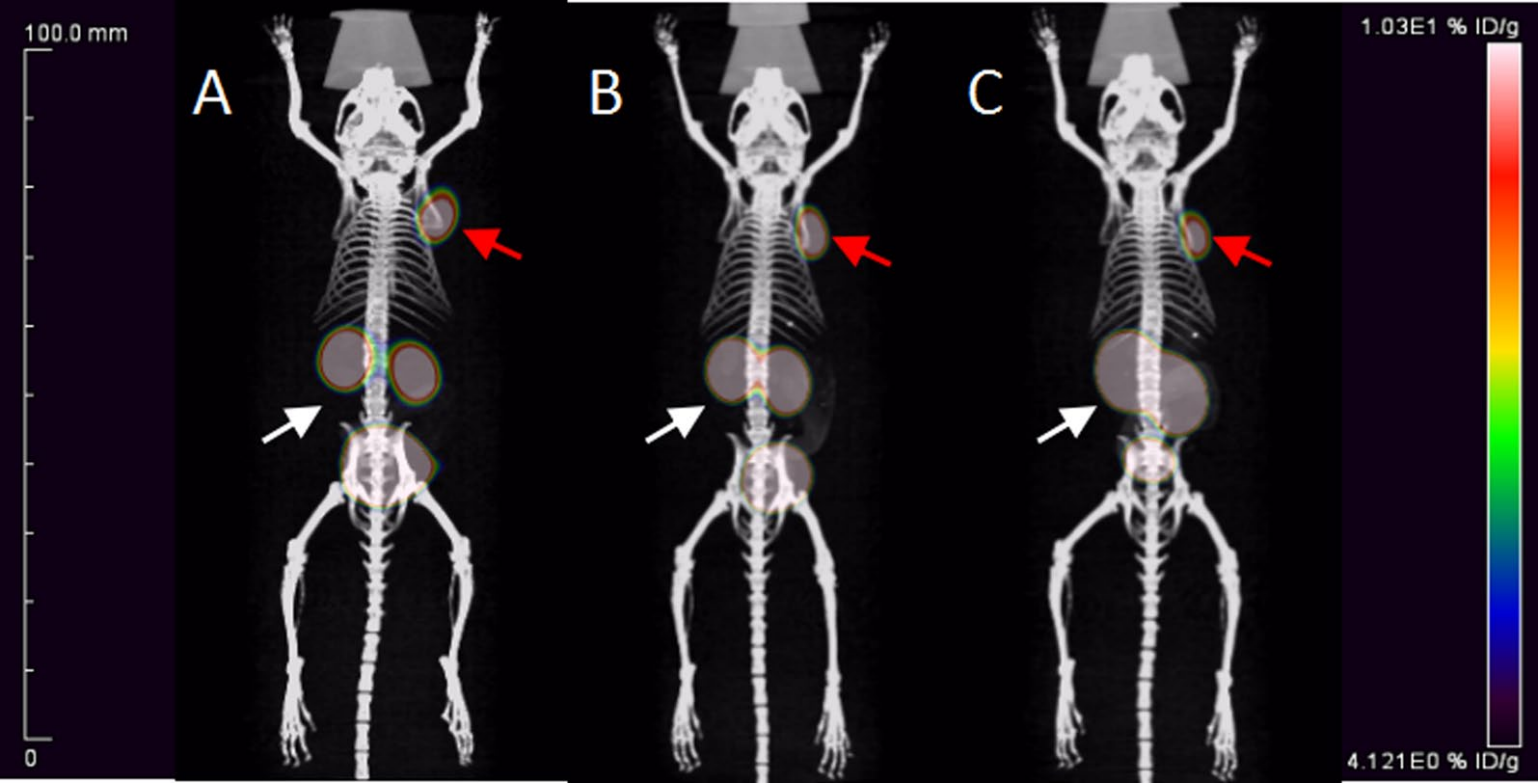
Maximum intensity projections of whole-body coronal micro-PET/CT images of a NOD/SCID male mouse bearing an LNCaP tumor xenograft (red arrow for the tumor, white arrow for the kidney). The tumor-targeting efficacy of [^68^Ga]Ga-PP4-WD, [^68^Ga]Ga-PP8-WD and [^68^Ga]Ga-Flu-1 was demonstrated by time-dependent static scans at 60 min p.i. of [^68^Ga]Ga-PP4-WD (**A**), [^68^Ga]Ga-PP8-WD (**B**), and [^68^Ga]Ga-Flu-1 (**C**). Approximately 2.6 MBq was injected into each mouse

Time-dependent static scans were performed for [^68^Ga]Ga-PP4-WD, [^68^Ga]Ga-PP8-WD, and [^68^Ga]Ga-Flu-1 at 10, 30, 60, and 120 min (Fig. 8, Additional file 1: Figure S6). These radioligands exhibited rapid accumulation in PSMA-positive LNCaP tumors as early as 10 min p.i., and by 120 min, all radioligands showed a clean background. Consistent with the biodistribution data, radioactivity for [^68^Ga]Ga-PP4-WD and [^68^Ga]Ga-PP8-WD was swiftly cleared from renal. In contrast, [^68^Ga]Ga-Flu-1demonstrated a significantly higher level of accumulated radioactivity in the renal area throughout the experiment.

**Fig. 9:**
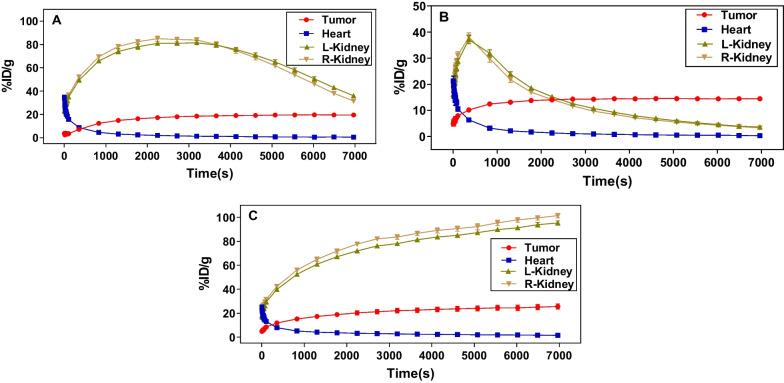
%ID/g (mean) was obtained from the whole-body coronal micro-PET/CT scans of the NOD/SCID male mice bearing LNCaP tumor xenografts. The tumor-targeting efficacies of [^68^Ga]Ga-PP4-WD (**A**), [^68^Ga]Ga-PP8-WD (**B**), and [^68^Ga]Ga-Flu-1 (**C**) were demonstrated by dynamic micro-PET scans

Following the static PET scan, a dynamic PET scan was performed to understand the pharmacokinetics of these radiotracers (Fig. 9). The dynamic uptake curves over a 2-h period revealed their fast-targeting properties, as the radiotracers quickly accumulated in the tumor and remained increasing uptake throughout the experiment. In terms of renal uptake, both [^68^Ga]Ga-PP4-WD and [^68^Ga]Ga-PP8-WD revealed an initial increase followed by a subsequent decrease. In contrast, the accumulation of [^68^Ga]Ga-Flu-1 exhibited a continually ascending pattern. Furthermore, both [^68^Ga]Ga-PP4-WD and [^68^Ga]Ga-PP8-WD displayed superior renal clearance compared with [^68^Ga]Ga-Flu-1. Dynamic coronal fused micro-PET/CT images obtained after injection of [^68^Ga]Ga-PP4-WD (A), [^68^Ga]Ga-PP8-WD (B), and [^68^Ga]Ga-Flu-1(C) in LNCaP tumor model over 2 h were performed in supplementary information (Additional file 1: Figure S7).

### Micro-SPECT/CT imaging

Balb/c-nu mice carrying PC3-PIP tumors were chosen for the micro-SPECT/CT imaging investigation of [^177^Lu]Lu-PP4-WD and [^177^Lu]Lu-PP8-WD (see Additional file 1: Figure S8). The findings indicated that, under the same parameters. Both radioligands exhibited rapid targeted uptake and maintained a favorable tumor-to-background ratio for up to 168 h, with minimal observable uptake in non-target organs, except for the bladder.

## Discussion

In previous work, we developed a PSMA-targeted inhibitor called [^68^Ga]Ga-Flu-1, which utilized a Lys-urea-Glu backbone and demonstrated excellent specificity and affinity in vivo for PSMA. However, we also observed a significant disparity in uptake between the kidneys and prostate tumor, with the kidneys showing much higher levels of [^68^Ga]Ga-Flu-1. The elevated uptake in kidneys raises concern about its potential impact on renal function and its potential to hinder the precise detection of kidney metastases in the cases where such metastases are present. PEG chains were often used as linkers to improve the hydrophilicity and the circulation time of the radiotracer in blood, leading to diverse biodistribution of the radiotracer [28, 29]. The lengths of PEG chains might have significantly impact on various biological properties of the drug, including hydrophilicity [30], absorption or release [31], blood circulation, and targeting ability with a size-dependent pattern [32, 33]. Lee W et al. showed that a PEGylated antibody cleared much faster from the blood while maintaining tumor uptake compared to its non-PEGylated counterpart [34].In this study, the compounds with PEG chains containing four repeat units of middle size and eight repeat units of larger size were incorporated, and compared with non-PEGylated ligand, the in vitro and in vivo properties were examined.

The results revealed that introducing PEG chain had a noticeable impact on the physicochemical properties of the compound, leading to significant impact on its in vitro and in vivo properties. Specifically, the water solubility, as expected, was enhanced after PEG modification, as indicated by the decrease in LogD_7.4_ value from − 2.64 ± 0.25 for the unmodified [^68^Ga]Ga-Flu-1 to − 4.23 ± 0.26 for [^68^Ga]Ga-PP8-WD, demonstrating a considerable improvement in water solubility. Accordingly, biodistribution properties of both radiotracers have undergone significant alterations, such as renal uptake, in particular, reduced by a factor of 40 and 20 at 120 min p.i. compared to [^68^Ga]Ga-Flu-1, respectively. Radioactivity accumulation in other normal organs like liver, was slightly reduced as well. Statistical analysis revealed that the uptake of [^68^Ga]Ga-Flu-1 in LNCaP tumor was significantly higher than [^68^Ga]Ga-PP8-WD at 60 min p.i. (*P* < 0.05). However, there was no significant difference between [^68^Ga]Ga-PP4-WD and [^68^Ga]Ga-Flu-1 (P > 0.05). The renal uptake of both [^68^Ga]Ga-PP4-WD and [^68^Ga]Ga-PP4-WD was significantly lower than for [^68^Ga]Ga-Flu-1 (*P* < 0.05) at given time points. These results indicated that PEG-modified compounds can effectively facilitate the renal clearance and reduce their uptake in the kidneys, likely by reduced tubular reabsorption, decreased binding to renal transporters, or rapid kidney filtration of the radioligands.

Whole body coronal micro-PET/CT static images of NOD/SCID male mice carrying LNCaP tumor xenografts had a clean background and high image quality. Combined with the dynamic uptake profile, it is evident that [^68^Ga]Ga-PP4-WD and [^68^Ga]Ga-PP8-WD were metabolized via kidneys as evidenced by a rapid decline of radioactivity within 2 h. In addition, when considering the dynamic uptake curves and the ability to effectively block tumor visualization in mice with tumors, both [^68^Ga]Ga-PP4-WD and [^68^Ga]Ga-PP8-WD highlighted the excellent specificity and quick targeting property for PSMA. These findings align with biodistribution results. Therefore, the substitution of the linker group with PEG remained the targeting characteristics while significantly decreasing renal uptake of the radiotracers. Although there was a slight decrease in tumor uptake, this was offset by reduced uptake in normal organs. As a result, these radiotracers still achieved impressive T/N (tumor-to-normal) values and image contrast.

## Conclusion

In summary, we have successfully developed two [^68^Ga]Ga-labeled PSMA-targeted radiotracers featuring PEG-modified chains. These radiotracers have demonstrated their effectiveness in significantly reducing kidney uptake while maintaining the absorbed dose in tumors. Both radiotracers exhibited strong binding and internalization characteristics in vitro, displayed high specificity and affinity for PSMA in vivo. Notably, [^68^Ga]Ga-PP4-WD, in particular, holds promise as a potential new diagnostic PET tracer for prostate cancer.

### Supplementary Information

**Additional file 1.**. The synthesis, characterization, IC50 measurements, biodistribution data, and small animal PET/CT images of these radiotracers.

## Declarations

### Availability of data and materials

The datasets used and/or analyzed during the current study available from the corresponding author on reasonable request.
