# Supplementary material for: Exploring the impact of PEGylation on pharmacokinetics: a size-dependent effect of polyethylene glycol on prostate-specific membrane antigen inhibitors
Source: EJNMMI Res. 2024 Feb 7;14:15. doi: 10.1186/s13550-024-01071-z (PMC10850047; doi:10.1186/s13550-024-01071-z)
Supplement: Supplementary file 1 — Additional file 1.. The synthesis, characterization, IC50 measurements, biodistribution data, and small animal PET/CT images of these radiotracers. [file 13550_2024_1071_MOESM1_ESM.docx]

**Exploring the impact of PEGylation on pharmacokinetics: a size-dependent effect of polyethylene glycol on prostate specific membrane antigen inhibitors**

Yang Liu ^1,2,3^, Li Xia ^1,2,4^, Haiyang Li^1,2,4^, Ping Cai ^1,2,4^, Sufan Tang^1,2,4^, Yue Feng ^1,2,3^, Guangfu Liu ^1,3^, Yue Chen ^1,2,3,^*, Nan liu^5^*, Wei Zhang^5*^, Zhijun Zhou ^1,2,3,4,^*

^1^ Department of Nuclear Medicine, the Affiliated Hospital, Southwest Medical University, Jiangyang District, Luzhou, Sichuan, China

^2^ Nuclear Medicine and Molecular Imaging Key Laboratory of Sichuan Province, Department of Nuclear Medicine, the Affiliated Hospital, Southwest Medical University, Jiangyang District, Luzhou, Sichuan, China

^3^ Institute of Nuclear Medicine, Southwest Medical University, Jiangyang District, Luzhou, Sichuan, China

^4^ Department of Pharmaceutics, School of Pharmacy, Southwest Medical University, Jiangyang District, Luzhou, Sichuan, China

^5^ Department of Nuclear Medicine, Sichuan Provincial People’s Hospital, University of Electronic Science and Technology of China, Sichuan, Chengdu, China

*Correspondence: [zhouzjiang@gmail.com](mailto:zhouzjiang@gmail.com), [liunan_815@163.com](mailto:liunan_815@163.com), zhangwscd@uestc.edu.cn, [chenyue5523@126.com](mailto:chenyue5523@126.com)

**Precursor Synthesis**

**1. The synthesis of s3**

To a solution of **s1**(1.15g,2.358mmol), **s2**(250mg,2.83mmol) in MeOH(10ml) and CH_3_COOH was added by drops and stirred at room temperature for 2h. NaBH_4_ (134mg, 3.537mmol) was added the above solution at 0℃, then moved to room temperature and stirred for 6 h. The mixture was poured in to NaHCO_3_ and extracted with EtOAc, dried over Na_2_SO_4_. The organic layer was concentrated in vacuo and purified by column chromatography (DCM/MeOH = 40/ 1) to give **s3** (877mg, 66.7% yield) as a colorless oil.

**2. The synthesis of s5**

**s4**(250mg, mmol) was dissolved in SOCl_2_(1ml) and stirred at room temperature for 24h. Solvent was evaporated under reduced pressure and the residue was redissolved in DCM(4ml). **s3**(430mg,0.768mmol) and Et_3_N (94mg,0.922mmol) was added to above solution and stirred at room temperature for 6h. The mixture was poured into ice water and extracted with DCM, washed with brine and dried over Na_2_SO_4._ The organic layer was concentrated in vacuo and purified by column chromatography (PE/EA= 3/1) to give **s5** (420mg, 72.7% yield) as a yellow oil.

**3. The synthesis of s6**

**s5**(300mg,0.399mmol) was dissolved in MeOH(5ml), then a solution of LiOH (20mg/ml,5ml) was added and the mixture was stirred at room temperature for 12h. The organic layer was concentrated in vacuo and the remaining mixture was diluted with water, 1M HCl was adjusted the PH to 5-6, then extracted with EA and dried over Na_2_SO_4._ The organic layer was concentrated under vacuo to provide the crude product **s6**(187mg, white solid), which was used in the next step without further purification.

1. **The synthesis of s9**

To a solution of **s7**(45mg, 0.0785mmol) in DMF (3mL) was added HATU (35.8mg, 0.0942mmol) and stirred at room temperature for 1 h. **s8** (54.8mg, 0.086mmol) and Et3N (2.8mg,0.027mmol) was added the above solution and stirred at room temperature for 9h. The mixture was poured into ice water and extracted with EA, washed with brine and dried over Na_2_SO_4_.The organic layer was concentrated in vacuo and purified by column chromatography (PE/EA= 5/1) to give the intermediate products (32mg, 45.5% yield). Then the intermediate product was dissolved in THF(2ml), 25%DEA was added at room temperature, the reaction was monitored by ESI-MS. When the reaction is over, the mixture was poured into ice water and extracted with DCM, washed with brine and dried over Na_2_SO_4._ The organic phase was concentrated under vacuo to provide the crude product **s9**, which was used in the next step without further purification.

**5. The synthesis of s10**

**s6**(20mg, 0.027mmol) and HATU (11.4mg, 0.03mmol) was dissolved in DMF(1.5ml) and stirred at room temperature for 1h. Followed by the addition of DIPEA (5.3mg, 0.041mmol) and **s9**(27.5mg, 0.03mmol), the mixture was stirred overnight at room temperature. When the reaction is over, the mixture was poured into ice water and extracted with EA, washed with brine and dried over Na_2_SO_4._ The organic phase was concentrated under vacuo to provide the intermediate product. Then, intermediate product was dissolved in 2mL 50% TFA/DCM solvent mixture and the reaction solution was stirred at room temperature overnight. The organic solvent and trifluoroacetic acid were removed by spin evaporation under reduced pressure, and the product was re-dissolved with acetonitrile and purified by preparative HPLC to obtain the product **s10**.

PP4-WD is synthesized in the same way as PP8-WD.

**Mass Spectral Analysis of the key compounds**

**1.PP4-WD**


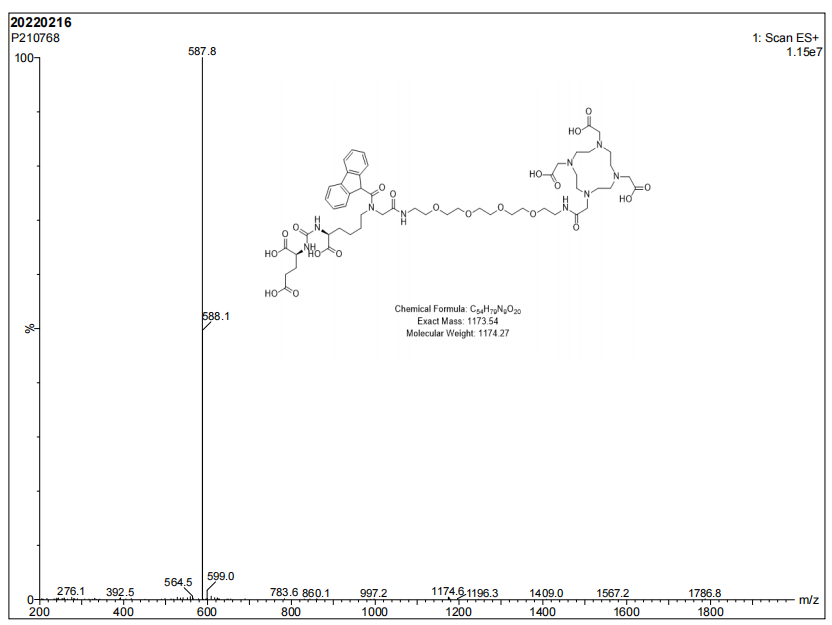


Figure S1. Mass Spectroscopy of PP4-WD (ESI+). Spectral data for PP4-WD: ESI-MS: calculated [M+H] ^+^ for C_54_H_80_N_9_O_20_ 1175.2765; found 1/2[M+H] ^+^ 587.8.

**2.PP8-WD**


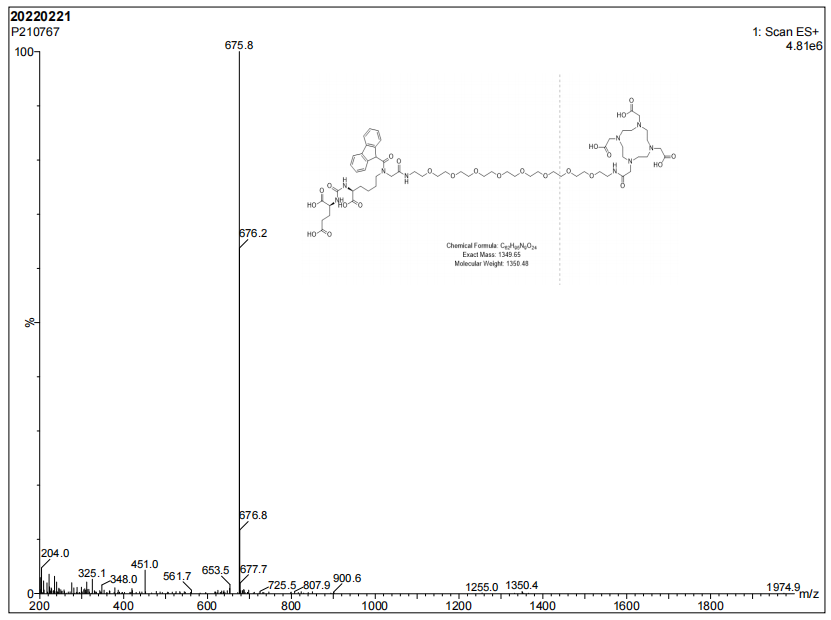


Figure S2. Mass Spectroscopy of PP8-WD (ESI+). Spectral data for PP8-WD: ESI-MS: calculated [M+H] ^+^ for C_62_H_96_N_9_O_24_ 1351.4885; found 1/2[M+H] ^+^ 675.8.

**Cell Affinity Studies**


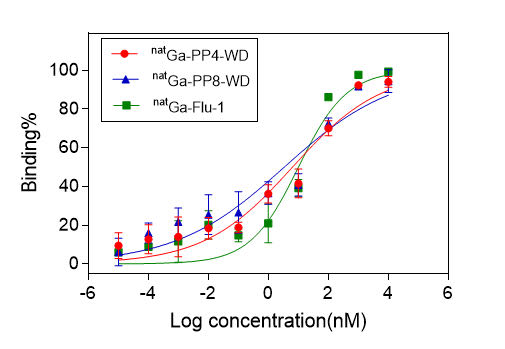


Figure S3. Representative inhibition curves of ^nat^Ga-PP4-WD ，^nat^Ga-PP8-WD and ^nat^Ga-Flu-1.

**Biodistribution**


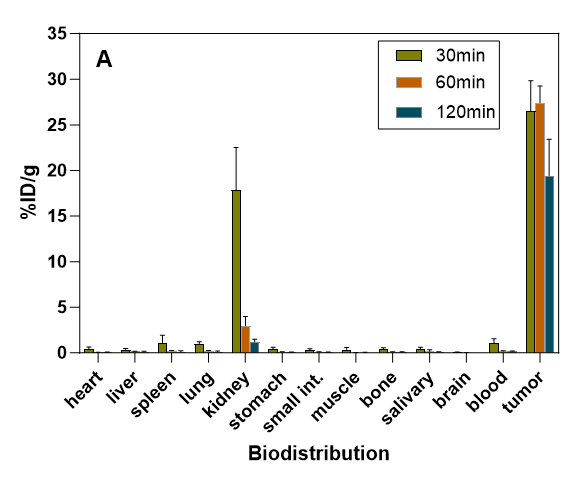

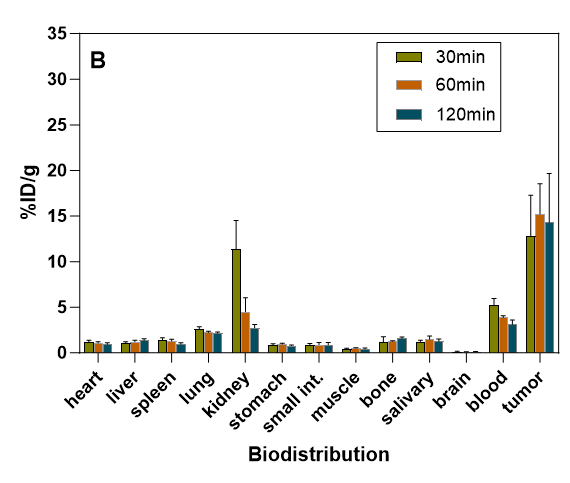


Figure S4. Organ biodistribution of [^68^Ga]Ga-PP4-WD (A) and [^68^Ga]Ga-PP8-WD (B) in PC3-PIP tumor model, the values expressed as %ID/g tissue at 30, 60, and 120 min post-injection (p.i.). Data are expressed as the mean ± SD (n = 5). small int. = small intestine.

Table S1. Organ biodistribution of [^68^Ga]Ga-PP4-WD in LNCaP tumor model as %ID/g tissue at 30 min, 60min, 120min post-injection. Data are expressed as mean ± SD (n=5).

| Tissue | 30min | 60min | 120min |
| --- | --- | --- | --- |
| Heart | 1.07±0.12 | 0.42±0.22 | 0.13±0.03 |
| Liver | 0.95±0.05 | 0.66±0.29 | 0.30±0.05 |
| Spleen | 2.45±0.20 | 0.82±0.54 | 0.32±0.05 |
| Lung | 3.49±0.27 | 1.14±0.16 | 0.42±0.11 |
| Kidney | 47.24±3.68 | 21.63±4.03 | 2.23±0.58 |
| Stomach | 1.38±0.58 | 0.39±0.20 | 0.13±0.01 |
| Small int | 2.23±0.23 | 0.43±0.17 | 0.15±0.07 |
| Muscle | 0.76±0.06 | 0.28±0.00 | 0.12±0.04 |
| Bone | 0.36±0.09 | 0.39±0.22 | 0.26±0.06 |
| Salivary | 3.72±0.67 | 1.40±0.22 | 0.44±0.06 |
| Brain | 0.12±0.04 | 0.06±0.03 | 0.03±0.01 |
| Blood | 3.30±0.22 | 0.84±0.11 | 0.28±0.08 |
| Tumor | 33.45±3.40 | 39.28±3.25 | 25.75±2.43 |

Table S2. Organ biodistribution of [^68^Ga]Ga-PP8-WD in LNCaP tumor model as %ID/g tissue at 30 min, 60min, 120min post-injection. Data are expressed as mean ± SD (n=5).

| Tissue | 30min | 60min | 120min |
| --- | --- | --- | --- |
| Heart | 1.31±0.23 | 0.59±0.31 | 0.23±0.01 |
| Liver | 1.53±0.77 | 0.79±0.44 | 0.41±0.09 |
| Spleen | 1.34±0.34 | 0.97±0.38 | 0.30±0.07 |
| Lung | 3.32±1.07 | 2.10±0.76 | 0.59±0.15 |
| Kidney | 25.63±3.46 | 15.25±2.59 | 6.39±1.56 |
| Stomach | 1.32±0.15 | 0.68±0.20 | 0.19±0.06 |
| Small int | 1.33±0.32 | 0.78±0.02 | 0.26±0.15 |
| Muscle | 1.06±0.12 | 0.58±0.24 | 0.21±0.14 |
| Bone | 0.24±0.12 | 0.37±0.13 | 0.01±0.00 |
| Salivary | 2.45±0.33 | 1.62±0.51 | 0.10±0.02 |
| Brain | 0.20±0.13 | 0.12±0.12 | 0.05±0.02 |
| Blood | 3.46±0.68 | 2.82±0.12 | 0.55±0.10 |
| Tumor | 16.18±2.53 | 18.64±2.20 | 17.12±2.57 |

| Tissue | 30min | 60min | 120min |
| --- | --- | --- | --- |
| Heart | 1.88±0.46 | 1.18±0.36 | 0.58±0.28 |
| Liver | 2.11±0.52 | 1.57±0.60 | 1.08±0.71 |
| Spleen | 9.45±5.92 | 7.25±3.39 | 2.38±1.75 |
| Lung | 5.77±2.01 | 3.27±0.84 | 1.23±0.47 |
| Kidney | 240.00±34.68 | 240.01±31.08 | 127.83±27.94 |
| Stomach | 1.84±0.31 | 0.86±0.11 | 0.42±0.17 |
| Small int | 2.52±0.60 | 1.89±1.10 | 0.46±0.25 |
| Muscle | 0.93±0.34 | 0.44±0.25 | 0.19±0.08 |
| Bone | 0.66±0.22 | 0.29±0.18 | 0.34±0.21 |
| Salivary | 5.84±3.65 | 3.02±1.27 | 1.46±1.05 |
| Brain | 0.18±0.10 | 0.11±0.10 | 0.06±0.04 |
| Blood | 5.53±2.11 | 4.69±2.33 | 3.07±2.7 |
| Tumor | 32.86±12.02 | 52.07±14.83 | 40.11±9.24 |

Table S3. Organ biodistribution of [^68^Ga]Ga-Flu-1 in LNCaP tumor model as %ID/g tissue at 30 min, 60min, 120min post-injection. Data are expressed as mean ± SD (n=5).

Table S4. The tumor-to-heart (T/H), tumor-to-liver (T/L), tumor-to-kidney (T/K), tumor-to-salivary (T/Sl) and tumor-to-blood (T/Bl) ratios were obtained from the biodistribution data of [^68^Ga]Ga-PP4-WD in LNCaP tumor model.

| T/NT | 30min | 60min | 120min |
| --- | --- | --- | --- |
| Tumor/Heart | 31.16±2.11 | 94.26±3.65 | 127.21±4.22 |
| Tumor/Liver | 35.03±3.31 | 59.23±2.56 | 84.59±3.21 |
| Tumor/Kidney | 0.71±0.12 | 1.50±0.20 | 11.54±2.32 |
| Tumor/Salivary | 11.00±0.99 | 28.0±1.45 | 58.00±2.45 |
| Tumor/Blood | 7.49±1.20 | 20.6±1.98 | 90.61±3.12 |

Table S5. The tumor-to-heart (T/H), tumor-to-liver (T/L), tumor-to-kidney (T/K), tumor-to-salivary (T/Sl) and tumor-to-blood (T/Bl) ratios were obtained from the biodistribution data of [^68^Ga]Ga-PP8-WD in LNCaP tumor model.

| T/NT | 30min | 60min | 120min |
| --- | --- | --- | --- |
| Tumor/Heart | 12.31±0.58 | 31.66±2.2 | 75.85±3.6 |
| Tumor/Liver | 10.58±0.32 | 23.74±1.90 | 41.69±2.9 |
| Tumor/Kidney | 0.63±0.11 | 1.22±0.35 | 2.68±0.22 |
| Tumor/Salivary | 5.23±0.24 | 11.50±1.19 | 45.05±2.56 |
| Tumor/Blood | 4.67±0.32 | 5.31±0.54 | 30.88±1.36 |

Table S6. The tumor-to-heart (T/H), tumor-to-liver (T/L), tumor-to-kidney (T/K), tumor-to-salivary (T/Sl) and tumor-to-blood (T/Bl) ratios were obtained from the biodistribution data of [^68^Ga]Ga-Flu-1 in LNCaP tumor model.

| T/NT | 30min | 60min | 120min |
| --- | --- | --- | --- |
| Tumor/Heart | 17.48±3.23 | 44.13±4.22 | 69.16±5.69 |
| Tumor/Liver | 15.57±2.89 | 33.17±3.09 | 37.14±3.65 |
| Tumor/Kidney | 0.14±0.12 | 0.22±0.21 | 0.31±0.21 |
| Tumor/Salivary | 5.63±1.11 | 17.24±3.54 | 27.47±4.33 |
| Tumor/Blood | 5.94±1.41 | 11.1±1.97 | 13.07±2.56 |

Table S7. Organ biodistribution of [^68^Ga]Ga-PP4-WD in PC3-PIP tumor model as %ID/g tissue at 30 min, 60min, 120min post-injection. Data are expressed as mean ± SD (n=5).

| Tissue | 30min | 60min | 120min |
| --- | --- | --- | --- |
| Heart | 0.39±0.26 | 0.06±0.01 | 0.06±0.03 |
| Liver | 0.36±0.14 | 0.16±0.03 | 0.14±0.05 |
| Spleen | 1.06±0.89 | 0.22±0.05 | 0.13±0.10 |
| Lung | 0.99±0.22 | 0.23±0.05 | 0.13±0.07 |
| Kidney | 17.85±4.66 | 2.96±1.04 | 1.21±0.30 |
| Stomach | 0.44±0.18 | 0.09±0.02 | 0.06±0.03 |
| Small int | 0.33±0.15 | 0.1±0.04 | 0.07±0.03 |
| Muscle | 0.36±0.25 | 0.04±0.02 | 0.04±0.03 |
| Bone | 0.41±0.17 | 0.11±0.02 | 0.08±0.03 |
| Salivary | 0.46±0.16 | 0.17±0.09 | 0.08±0.03 |
| Brain | 0.05±0.04 | 0.02±0.01 | 0.01±0.01 |
| Blood | 1.07±0.47 | 0.19±0.05 | 0.17±0.04 |
| Tumor | 26.49±3.34 | 27.43±1.81 | 19.42±4.00 |

Table S8. Organ biodistribution of [^68^Ga]Ga-PP8-WD in PC3-PIP tumor model as %ID/g tissue at 30 min, 60min, 120min post-injection. Data are expressed as mean ± SD (n=5).

| Tissue | 30min | 60min | 120min |
| --- | --- | --- | --- |
| Heart | 1.21±0.19 | 1.09±0.12 | 0.94±0.16 |
| Liver | 1.11±0.10 | 1.24±0.16 | 1.38±0.19 |
| Spleen | 1.39±0.26 | 1.30±0.21 | 0.96±0.14 |
| Lung | 2.64±0.23 | 2.25±0.13 | 2.16±0.14 |
| Kidney | 11.38±3.15 | 4.52±1.51 | 2.74±0.38 |
| Stomach | 0.83±0.17 | 0.98±0.09 | 0.79±0.08. |
| Small int | 0.87±0.18 | 0.92±0.23 | 0.89±0.27 |
| Muscle | 0.45±0.06 | 0.52±0.07 | 0.43±0.11 |
| Bone | 1.21±0.57 | 1.26±0.07 | 1.62±0.13 |
| Salivary | 1.23±0.17 | 1.49±0.37 | 1.26±0.27 |
| Brain | 0.13±0.05 | 0.12±0.02 | 0.13±0.03 |
| Blood | 5.2±0.77 | 3.89±0.18 | 3.12±0.48 |
| Tumor | 12.81±4.48 | 15.21±3.33 | 14.3±5.37 |

**Micro‑PET/CT and Micro‑SPECT/CT imaging**


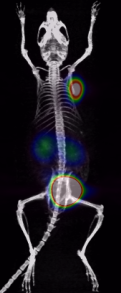

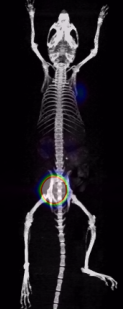

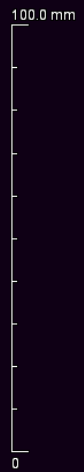

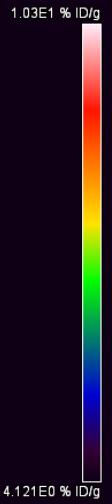


tumor


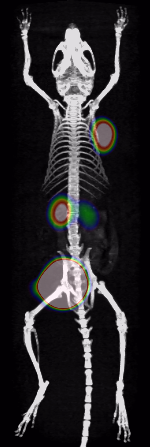

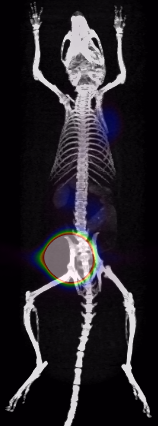

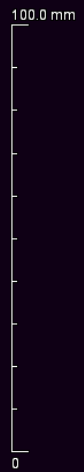

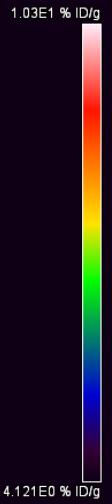


tumor

A

B

[^68^Ga]^68^Ga-PP4-WD blocking by-2-PMPA [^68^Ga]^68^Ga-PP8-WD blocking by-2-PMPA


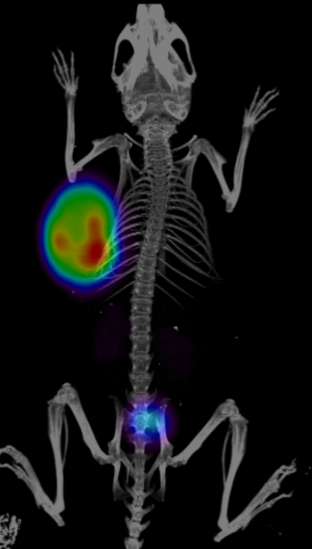

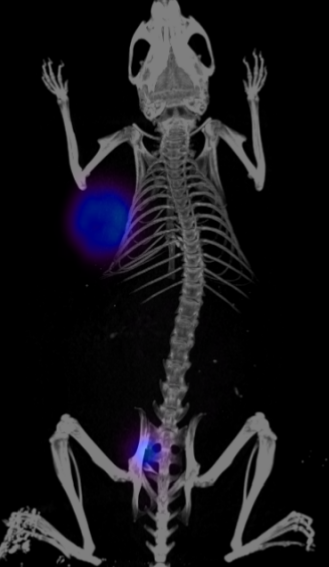

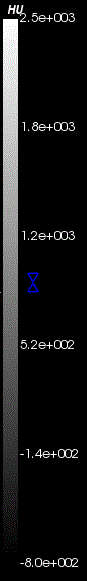

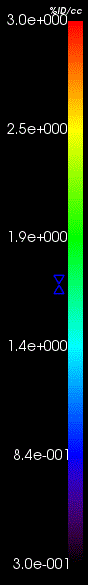


tumor


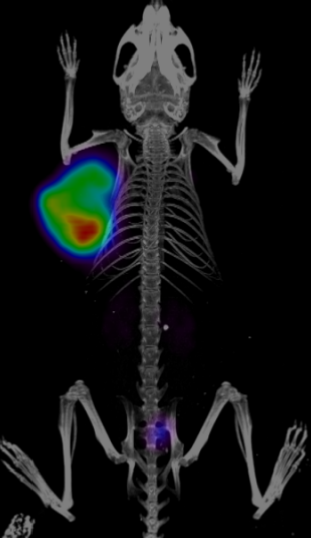

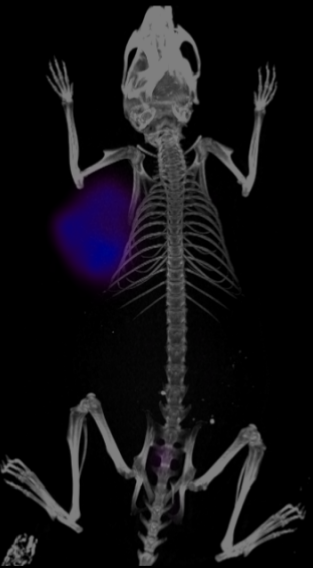

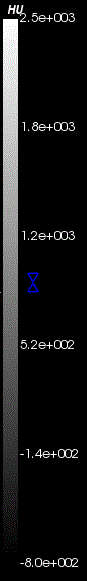

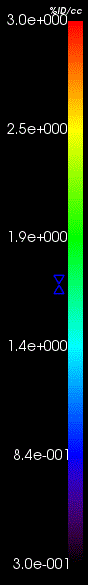


tumor

C

D

[^68^Ga]^68^Ga-PP4-WD blocking by-2-PMPA [^68^Ga]^68^Ga-PP8-WD blocking by-2-PMPA

Figure S5. Micro-PET/CT images of [^68^Ga]Ga-PP4-WD (A) and [^68^Ga]Ga-PP8-WD (B) at 1 h post-injection using NOD/SCID male mouse bearing LNCaP tumor xenograft. Micro-PET/CT images of [^68^Ga]Ga-PP4-WD (C) and [^68^Ga]Ga-PP8-WD (D) at 1 h post-injection using balb/c-nu male mouse bearing PC3-PIP tumor xenograft. Left is static scans at 1h, approximately 2.6 MBq was injected into each mouse, right is blocking scan, 40 nmol 2-PMPA was injected into each mouse before the radiolabeled drug was injected.


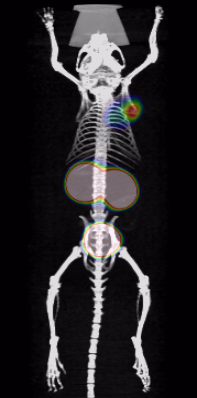

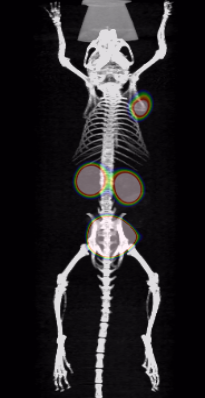

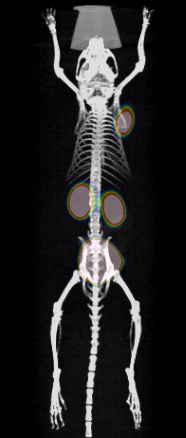

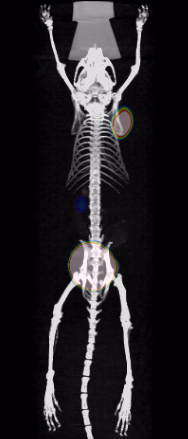

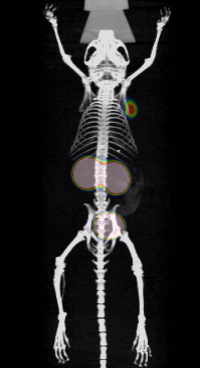

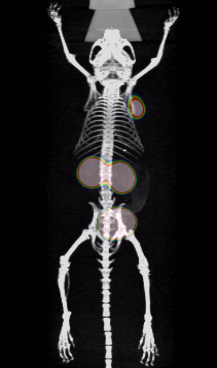

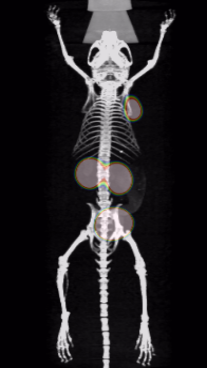

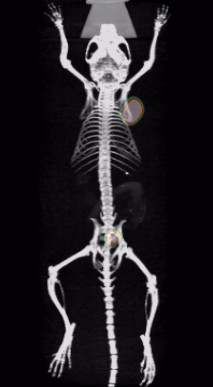

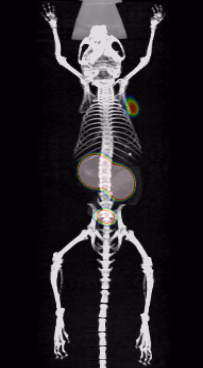

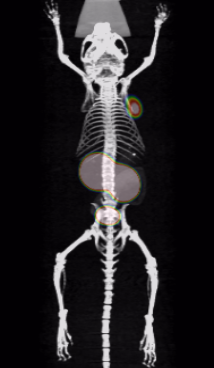

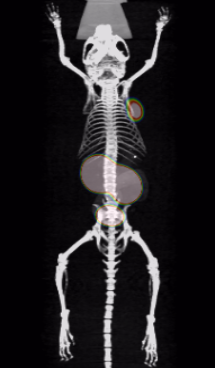

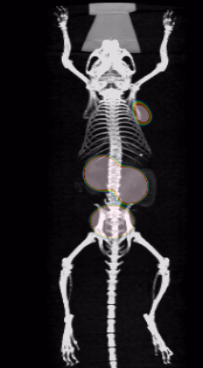

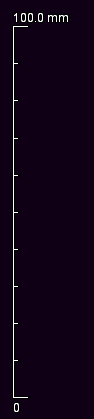

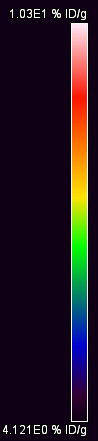

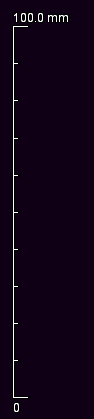

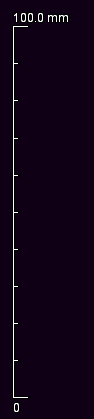

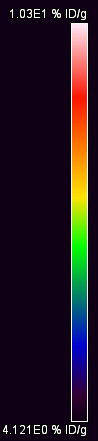

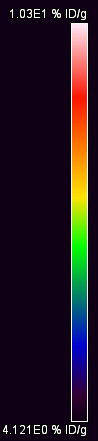


A

B

C

Figure S6. Maximum intensity projections of whole-body coronal micro-PET/CT images of a NOD/SCID male mouse bearing an LNCaP tumor xenograft (red arrow for the tumor, white arrow for the kidney). The tumor-targeting efficacy of [^68^Ga]Ga-PP4-WD, [^68^Ga]Ga-PP8-WD and [^68^Ga]Ga-Flu-1 was demonstrated by time-dependent static scans at 10, 30, 60, and 120 min (from left to right) post-injection of [^68^Ga]Ga-PP4-WD (A), [^68^Ga]Ga-PP8-WD (B) and [^68^Ga]Ga-Flu-1 (C). Approximately 2.6 MBq was injected into each mouse.


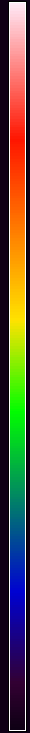

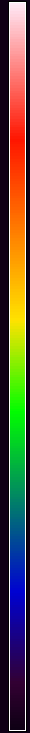


1min 2min 10min 25min 50min 60min 80min 100min 120min


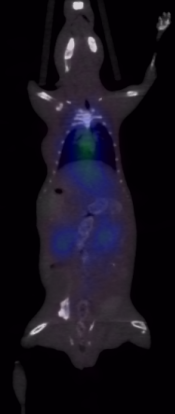

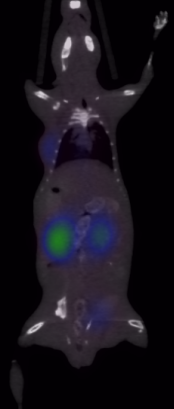

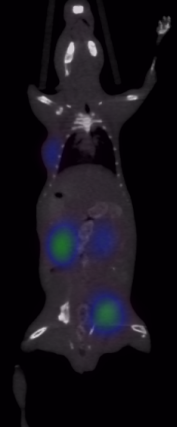

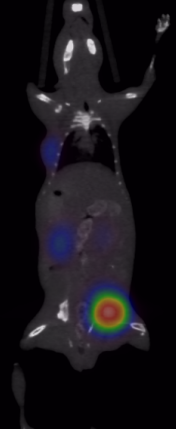

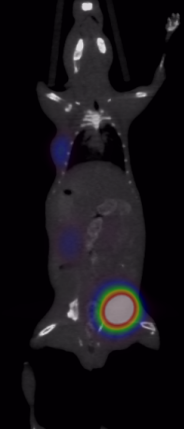

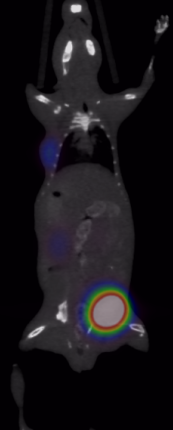

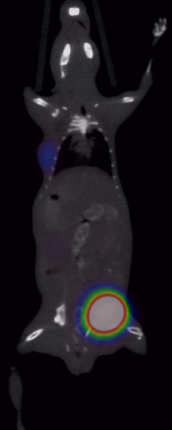

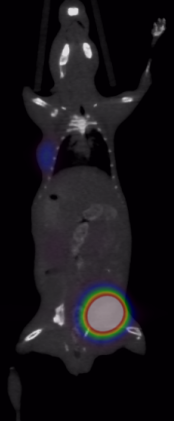

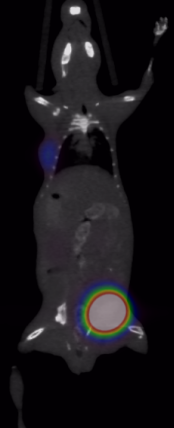

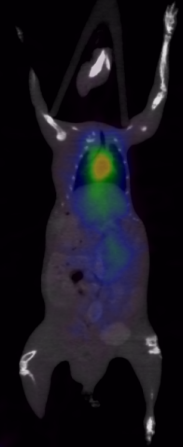

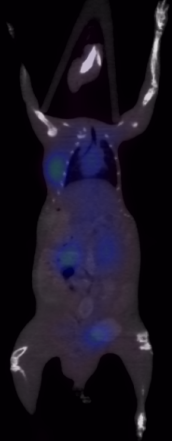

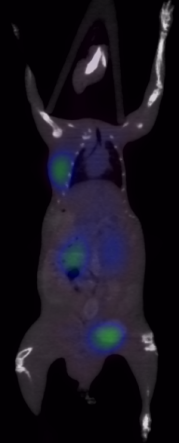

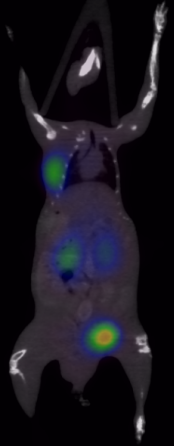

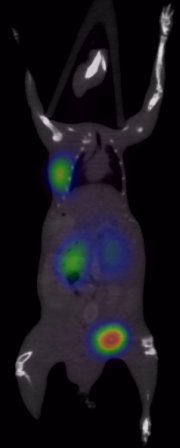

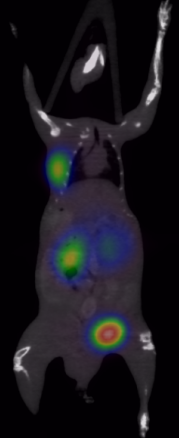

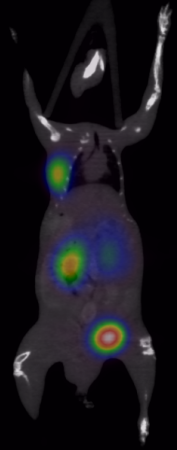

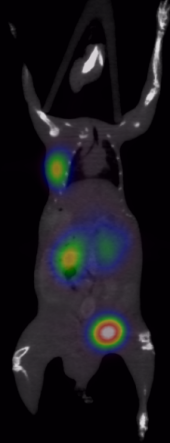

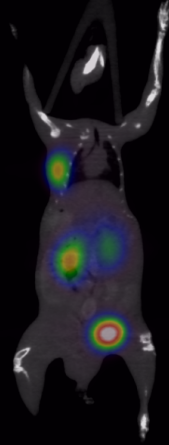

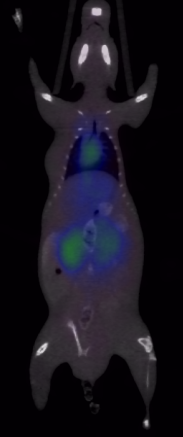

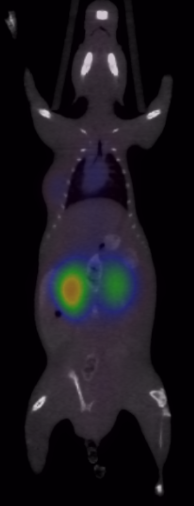

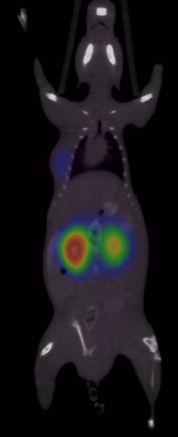

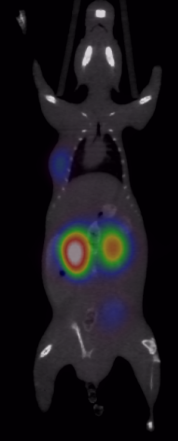

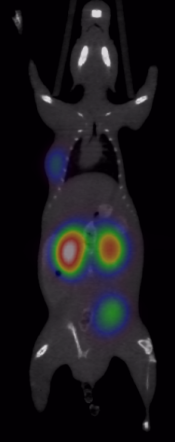

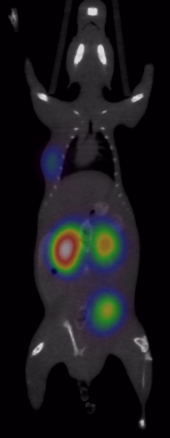

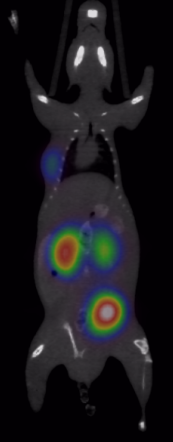

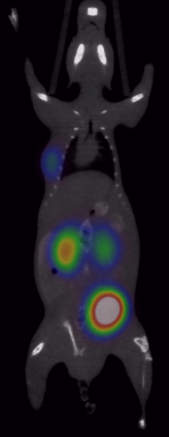

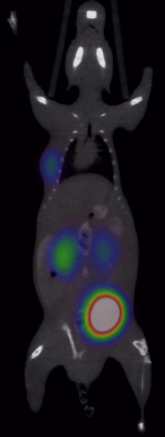

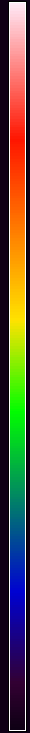

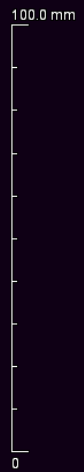

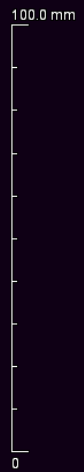

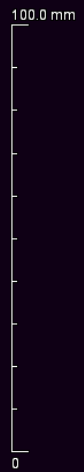


tumor

tumor

tumor

A

C

B

Figure S7. Dynamic coronal fused micro-PET/CT images obtained after injection of [^68^Ga]Ga-PP4-WD (A), [^68^Ga]Ga-PP8-WD (B), and [^68^Ga]Ga-Flu-1(C) in LNCaP tumor model over 2 h .

1h 4h 24h 48h 72h 96h 120h 144h 168h


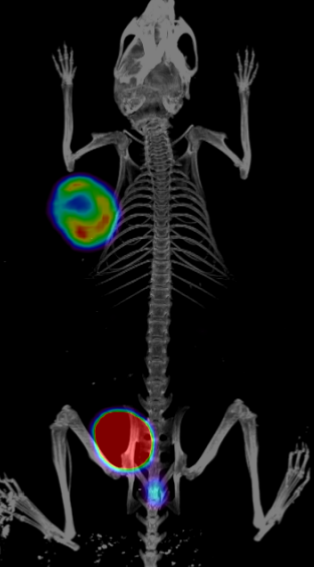

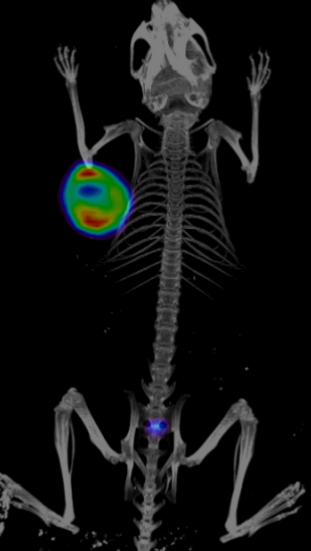

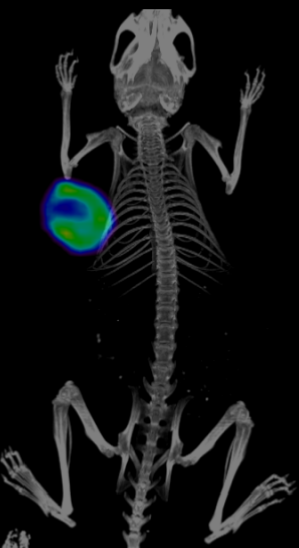

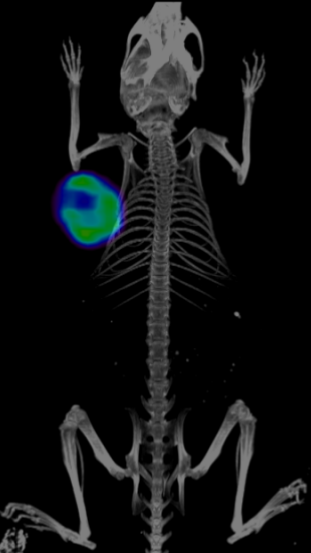

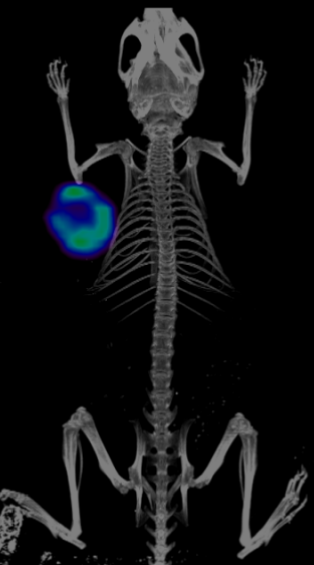

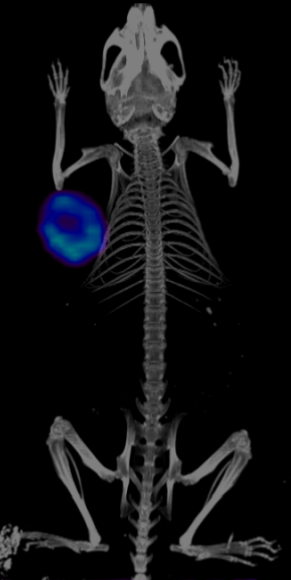

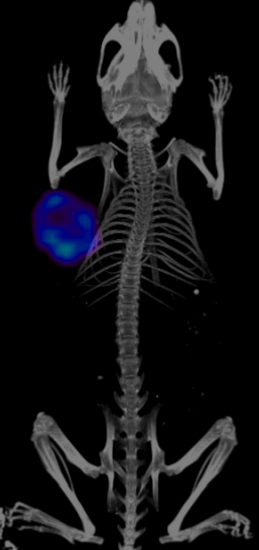

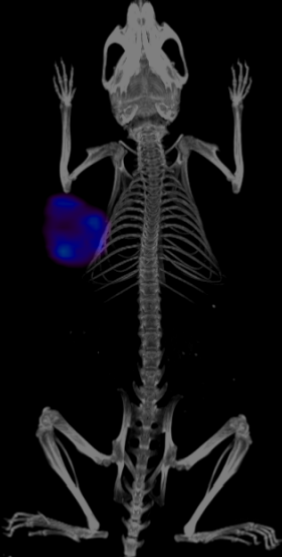

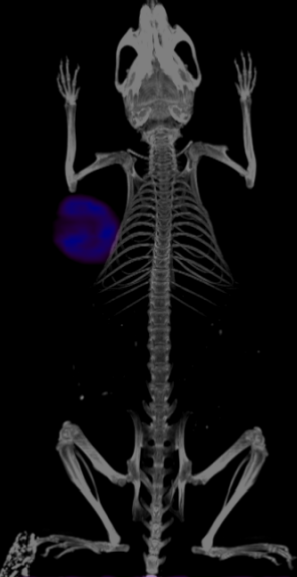


**A**


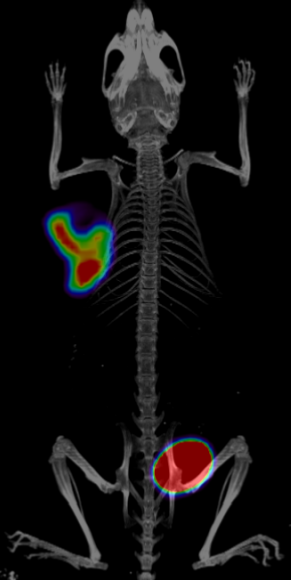

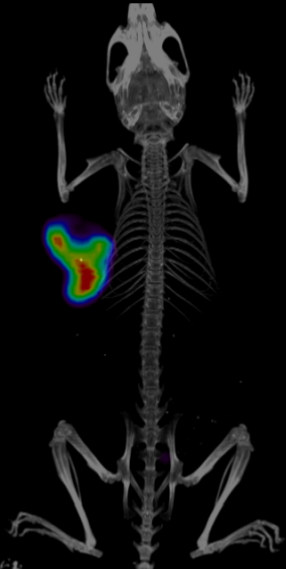

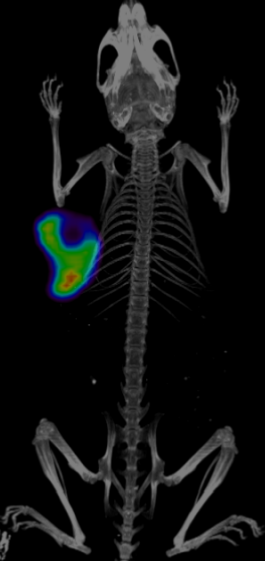

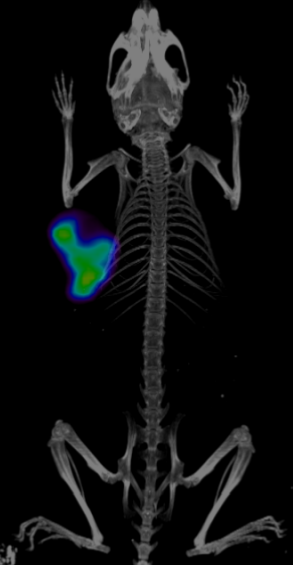

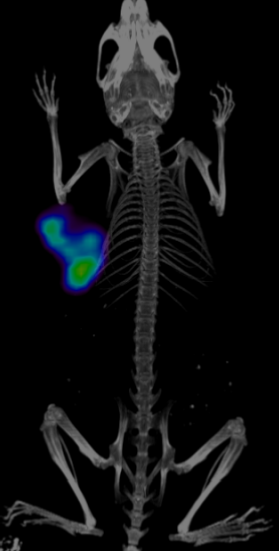

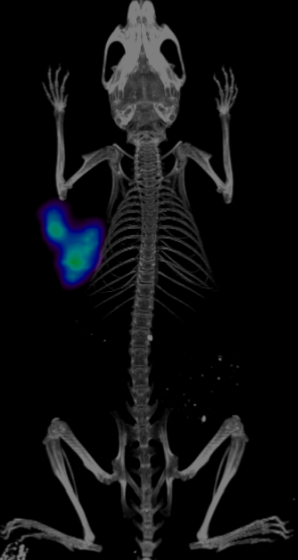

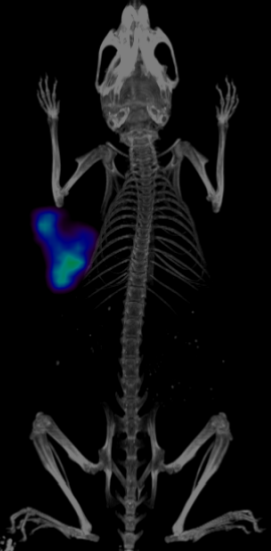

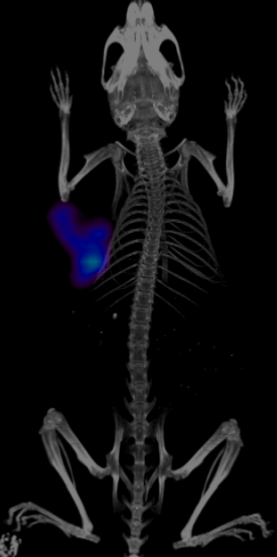

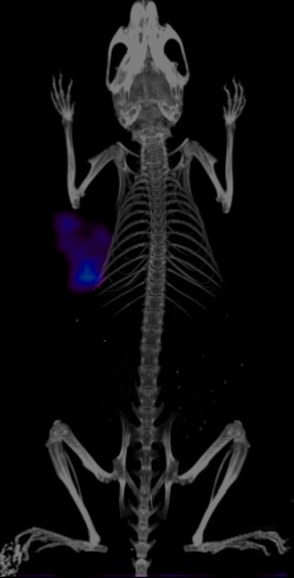


**B**


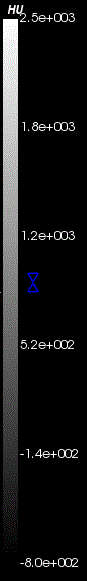

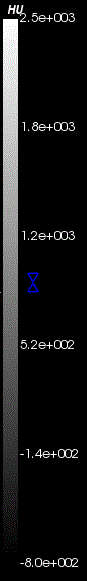


tumor


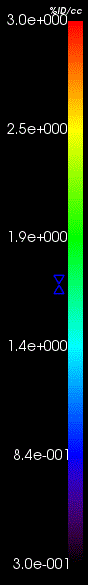

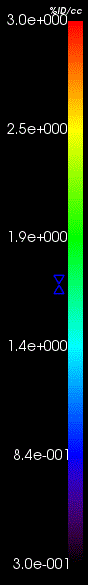


tumor

Figure S8. Maximum intensity projections of whole-body coronal micro-PET/CT images of a balb/c-nu male mouse bearing an PC3-PP tumor xenograft . The tumor-targeting efficacy of [^177^Lu]Lu-PP4-WD and [^177^Lu]Lu-PP8-WD was demonstrated by time-dependent static scans at 1h, 4h, 24h, 48h, 72h, 96h, 120h, 144h and 168h (from left to right) post-injection of [^177^Lu]Lu-PP4-WD (A) and [^177^Lu]Lu-PP8-WD (B) . Approximately 7.4 MBq was injected into each mouse.

tumor

tumor

tumor
